# High-Temperature-Induced Defects in Tomato (*Solanum lycopersicum*) Anther and Pollen Development Are Associated with Reduced Expression of B-Class Floral Patterning Genes

**DOI:** 10.1371/journal.pone.0167614

**Published:** 2016-12-09

**Authors:** Florian Müller, Jiemeng Xu, Lieke Kristensen, Mieke Wolters-Arts, Peter F. M. de Groot, Stuart Y. Jansma, Celestina Mariani, Sunghun Park, Ivo Rieu

**Affiliations:** 1 Department of Molecular Plant Physiology, Institute for Water and Wetland Research, Radboud University, Nijmegen, The Netherlands; 2 Department Horticulture and Human Health, Kansas State University, Manhattan, Kansas, United States of America; University of Tsukuba, JAPAN

## Abstract

Sexual reproduction is a critical process in the life-cycle of plants and very sensitive to environmental perturbations. To better understand the effect of high temperature on plant reproduction, we cultivated tomato (*Solanum lycopersicum)* plants in continuous mild heat. Under this condition we observed a simultaneous reduction in pollen viability and appearance of anthers with pistil-like structures, while in a more thermotolerant genotype, both traits were improved. Ectopic expression of two pistil-specific genes, *TRANSMITTING TISSUE SPECIFIC* and *TOMATO AGAMOUS LIKE11*, in the anthers confirmed that the anthers had gained partial pistil identity. Concomitantly, expression of the B-class genes *TOMATO APETALA3*, *TOMATO MADS BOX GENE6* (*TM6*) and *LePISTILLATA* was reduced in anthers under continuous mild heat. Plants in which *TM6* was partially silenced reacted hypersensitively to temperature elevation with regard to the frequency of pistilloid anthers, pollen viability and pollen quantity. Taken together, these results suggest that high-temperature-induced down-regulation of tomato B-class genes contributes to anther deformations and reduced male fertility. Improving our understanding of how temperature perturbs the molecular mechanisms of anther and pollen development will be important in the view of maintaining agricultural output under current climate changes.

## Introduction

Sexual reproduction is a key process in the life-cycle of plants, both in natural and in agricultural settings, with fitness and yield, respectively, depending directly on its success. The efficiency of the process, however, is strongly influenced by environmental conditions, such as water availability and ambient temperature [1,2]. With the current, rapid changes in global climate, the average and maximum temperatures are expected to increase in many parts of the world as well as the frequency of heat waves [3]. Studies in various plant species, monocot and dicot, have shown that development and functioning of especially the male gametophyte is highly sensitive to both continuous mild increases and short extremes in temperature [4,5]. A number of studies have addressed the problem of heat-induced male infertility in plants and identified deviations in various tissues [6–8] and metabolic processes [9–16]. During their development, the male gametophytes are actively supported by the surrounding sporophytic cell layer, called the tapetum [17]. Because developmental defects in the pollen under high temperature are often accompanied by developmental defects in the tapetum [7,8,18–23] it has been suggested that the the latter constitutes the primary defect [1,24]. The sensitivity of the tapetum to high temperature has been confirmed by a transcriptome analysis in rice, showing that heat-repressed genes in anthers preferentially locate to this tissue [16].

Patterning of floral organs is primarily under control of a set of MADS-box genes. These genes encode transcription factors with a conserved MADS-box DNA binding domain and are thought to exist in all eukaryotes. In plants, MADS-box genes are involved in controlling major aspects of development, including male and female gametophyte development, embryo and seed development, as well as root, flower and fruit development [25].The MADS-box genes involved in floral organ identity are also referred to as the ABC genes. The ABC model was proposed as a result of research on mutants with homeotic deformations [26]. The model divides the floral meristem into four whorls, each characterized by the expression of specific (combinations) of ABC genes. This, in turn leads to the formation of the four kinds of flower organs: activity of A-class genes alone in the first whorl will result in the formation of sepals, the combination of A- and B-class genes leads to petals in the second whorl, B- and C-class genes produce stamens in the third whorl and C-class genes alone direct pistil development in the central fourth whorl [25,27].

When examining tomato flowers grown under continuous mild heat conditions, we observed not only a reduction in pollen number and viability, but also the appearance of anther deformations resembling a partial conversion to pistil identity. The aim of this study was to identify the molecular changes underlying these deformations and determine the relation between anther deformation and impaired development of the male gametophyte under mildly elevated temperatures.

## Materials and Methods

### Plant material and treatments

*S*. *lycopersicum* cultivar Red Setter was obtained from Metapontum Agrobios (Metaponto, Italy). The cultivar Microtom (TOMJP00001) was provided by University of Tsukuba, Gene Research Center, through the National Bio-Resource Project (NBRP) of the MEXT, Japan. The *TM6-*RNAi line (TM6i6) in the Microtom background was kindly provided by Dr Vivian Irish (Yale University, New Haven, USA; De Martino et al., 2006). The lines *AtGRXS17-3* and *AtGRXS17-9* were in the Rubicon background [28].

Plants were raised in climate chambers (14 hours per day light, ~200 μmol/m^2^s at plant level, ~70% humidity) under control conditions (25°C day, 19°C night). Upon appearance of the first inflorescence, buds and flowers were removed and the plants were kept under control conditions, continuous mild heat (CMH32: 32°C day, 26°C night) or higher continuous mild heat (CMH34: 34°C day, 28°C night). Flowers that developed fully under these conditions were used to determine the frequency of anther deformations, light microscopy, gene expression studies and pollen germination rate. Pollen germination was analysed by *in-vitro* pollen germination as described by [29] with minor changes (sucrose and PEG400 were adjusted to 5% and 25%, respectively). To determine the frequency of anther deformations in Red Setter at least eight plants were used per treatment and all flowers produced over a period of two weeks were harvested (24 flowers under control conditions, 91 flowers under CMH32 and, due to reduced flower set, 10 flowers under CMH34). To analyse the lines *AtGRXS17-3* and *AtGRXS17-9* three plants were used per treatment and ten flowers per plant, that did fully develop under these conditions, were harvested. Flowers that did showed spacing between anthers, bent anther tips, green or shortened stamen were counted as deformed.

For gene expression studies seven and four plants of the cultivar Red Setter were grown under control and CMH32, respectively. Anthers and pistils of the flowers were harvested separately and sampled randomly into pools (5 mature flowers or 10 flower buds with an anther length of 2–3 mm per pool).

To study the Microtom *TM6*-RNAi line, Microtom wild type and transgenic plants were grown in climate cabinets (MC1600, Snijders Labs, The Netherlands) under control conditions (constant 22°C, 12 hours LED-light from Philips Green Power LED DR/B/FR 120, ~250 μmol/m^2^s, 60% humidity) and moved into milder continuous mild heat (CMH30: 30.5°C day, 25.5°C night,) upon flowering. To determine the rate of flower deformations newly opened flowers were harvested daily on day 8 to 15 after moving the plants into CMH30, flowers were scored as described above. Pollen germination was determined from newly opened flowers on day 8 after moving into CMH30 and pollen number on the days 13–15 after moving into CMH30. Flowers and pollen from control conditions were harvested and analysed on the same days. Pollen germination was analysed as described above and pollen number was determined with a Bürker-Türk counting chamber.

### Light microscopy

Anthers were fixed in FAA for 24 hours at 4°C, dehydrated in a graded ethanol series and embedded in Technovit 7100. Sections of 5–7μm were cut with a rotary microtome and stained with toluidine blue 0.1% in 1% borax. Sections were viewed and photographed with a Leitz Orthoplan microscope, equipped with a Leica camera DFC 420C.

### qRT-PCR

RNA was extracted from the flower organs using Trizol (Invitrogen) and treated with DNase I. cDNA was synthesized using the IScript kit (Bio-Rad) and the PCR (end volume of 25 μl, containing cDNA equivalent to 10 ng RNA) was performed using the SybrGreen iQ mix (Bio-Rad). The primer pairs (Table 1) used for the analysis were designed using Beacon Designer (Premier Biosoft). Cq values were determined by CFX Manager 3.0 (Bio-Rad) and the efficiency was calculated using LinReg PCR. The normalization factor was calculated using GeNorm (Biogazelle), using *SAND*, *CAC*, *EF1α* and *RPL8* as reference genes. The qRT-PCR was performed with four and five biological replicates from flower buds and mature flowers, respectively.

**Table 1:** List of primers used in this study.

Gene	Accession number	Sequence forward primer	Sequence reverse primer
*TAP3*	Solyc04g081000.2.1	ATATTAGACTTACGCCTTC	AATTACTACTCAACCTAGAG
*TM6*	Solyc02g084630.2.1	GTTCACAGTAATGGCGTTA	ATAATAGAGTGCTTAACACAGAA
*TPI*	Solyc06g059970.2.1	CCCACTTTAAATTAAGAACT	GCTAGGTAAGTAGAACAAT
*LePI*	Solyc08g067230.2.1	ATTGAGACAACTAGAGATAGCA	TAGATGGGAGGTTTGATTTAGA
*TAG1*	Solyc02g071730.2.1	CTCAGCAATTCGATACTC	CTCTCAAGCACATTAGAC
*TAGL1*	Solyc07g055920.2.1	GCATAGCAGAGGTAGAGA	TTACAGGCAGGAAGTTATTG
*SlTTS*	Solyc02g078100.2.1	AAGCCACCATCACCTTAT	TCAGCCTGTTCAACTAATG
*TAGL11*	solyc11g028020.1.1	TCTACTGAGGAGGAAGGAA	AAGTTGAGATGTCCAGAGTAT
*SAND*	Solyc03g115810.2.1	TTGCTTGGAGGAACAGACG	GCAAACAGAACCCCTGAATC
*CAC*	Solyc08g006960.2.1	CCTCCGTTGTGATGTAACTGG	ATTGGTGGAAAGTAACATCATCG
*EF1α*	Solyc06g005060.2.1	TGATCAAGCCTGGTATGGTTGT	CTGGGTCATCCTTGGAGTT
*RPL8*	Solyc10g006580.2.1	GGTGTTCTGGTGATTACGCCATTG	CCAGCAACCTGACCAATCATAGC

### Statistics

Frequencies of anther deformation under three temperatures and in three genotypes were averaged on plant level and compared using a non-parametric Kruskal-Wallis one-way ANOVA followed by Mann-Whitney U test to compare group means pairwise. As the data on frequencies of anther deformation in the TM6 experiment did not comply with assumptions of ANOVA, they were tested using a generalized linear model on count data. Pollen germination data and pollen number data were logit-transformed and log-transformed, respectively, and analysed by a one-way ANOVA with Tuckey or two-way ANOVA with Tuckey followed by a one-way ANOVA to test for differences between individual groups, as appropriate. For analysis of the qRT-PCR data, log-transformed normalized relative quantities were compared using a student’s t-test, according to [30]. All analyses were performed using IBM SPSS statistics version 21 (IBM).

## Results

### Co-occurrence of floral deformations and low pollen viability under continuous mild heat conditions

**Fig 1:**
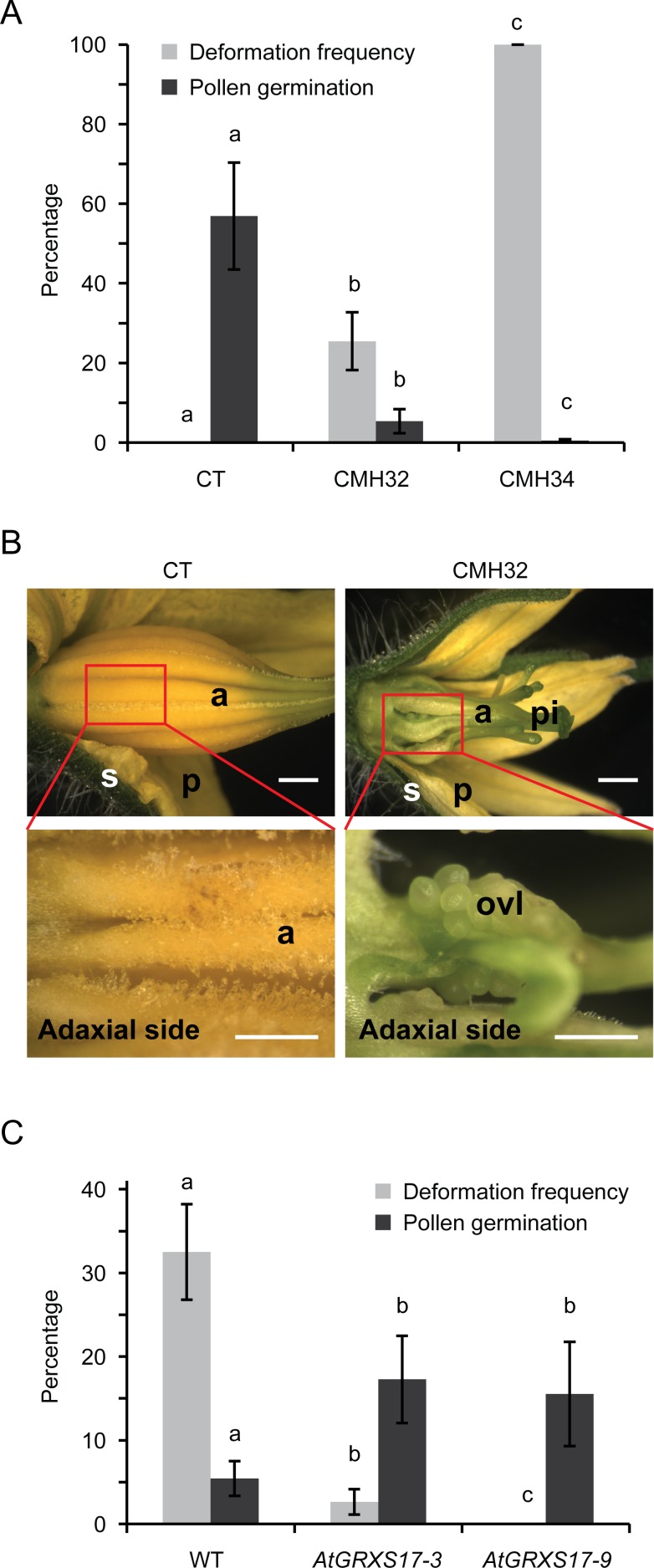
Continous mild heat conditions affect pollen viability and flower deformation simultaneously. A, Frequency of anther deformations and pollen germination rate of tomato plants (cv. Red Setter) grown under different temperature regimes (CT, CMH32, CMH34). Values represent the mean ± SD, different letters indicate statistically significant differences (per trait, P<0.01). B, Mature flowers of wild-type tomato (cv. Red Setter) from control (CT) and CMH32 conditions. Insets show the adaxial side of the indicated boxed regions. Scale bars: 1 mm and 0,5 mm (insets). a, anther; ovl, ovule-like structures; p, petal; pi, pistil; s, sepal. C, Frequency of anther deformations and pollen germination rate of wild type (WT) tomato plants (cv. Rubicon) and transgenic lines *AtGRXS17-3* and *AtGRXS17-9* grown under CMH32. Under control conditions, no anther deformations were observed, and percentage pollen viability was 60 ± 12 (SD), 60 ± 13 and 56 ± 9 for WT, *AtGRXS17-3* and *AtGRXS17-9*, respectively (no significant differences between genotypes). Values represent the mean ± SD, different letters indicate statistically significant differences (per trait, P<0.05).

As has been reported before [31], tomato pollen viability was compromised when plants were grown under contiunuous mild heat (CMH), with clear effects being visible at ~32°C/26°C day- and night-time temperatures (CMH32; Fig 1A). In addition, we observed that under these conditions part of the anthers developed deformations, consisting of spacing between anthers, twisting and greening of the tips (“antheridial cone splitting”; Fig 1B). The frequency of these deformations increased with higher temperatures (CMH34), while at the same time pollen viability was further reduced (Fig 1A). To test whether the negative correlation between the occurrence of anther deformation and pollen viability remained when using a different perturbation than temperature, we compared wild-type plants with plants with increased heat tolerance, due to overexpression of the glutaredoxin *GRXS17* [28]. When grown under control conditions, transgenic lines were indistinguishable from the wild type. In contrast, when grown at CMH32, the *AtGRXS17*-overexpressing lines showed significantly fewer flowers with anther deformations than the wild type, while the pollen viability was higher than in the wild type (Fig 1C). Thus, in both experimental systems, pollen viability and the presence of anther deformation were negatively correlated within the same temperature range.

### CMH-induced anther deformations are homeotic

**Fig 2:**
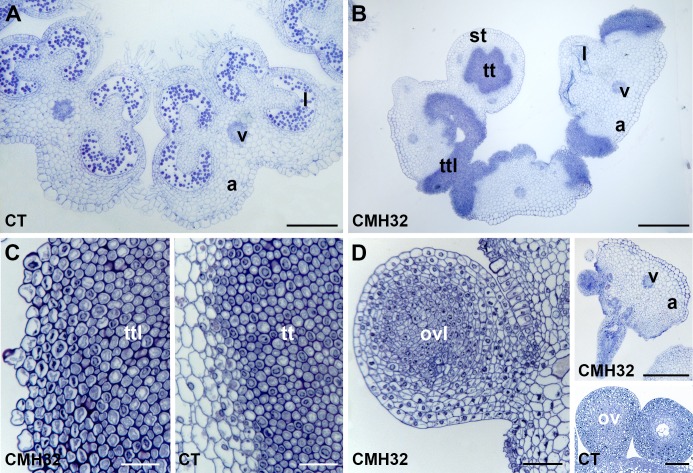
Cross sections of anthers and pistils from mature flowers of the tomato cultivar Red Setter grown under control and continuous mild heat conditions. A, overview of anthers grown under control conditions (CT). Scale bar: 300 μm. B, overview of anthers and pistil from CHM32. Scale bar 300 μm. C, transmitting-tissue-like cells from an anther grown under CMH32 (close up of B) on the left, and true stylar transmitting tissue from control conditions on the right. Scale bar 20 μm. D, ovule-like deformation from an anther grown under CMH32 (scale bar 30 μm). Upper inset shows overview of CMH32 anther with ovule-like deformation (scale bar 300 μm), lower inset shows a true ovule from control conditions (scale bar 50 μm). a, anther; l, locule; ov, ovule; ovl, ovule-like structure; st, style; tt, transmitting tissue; ttl, transmitting tissue-like cells; v, vascular bundle.

Closer inspection revealed that in some cases aberrant tissue was present on the adaxial side of the anthers grown under CMH32 (Fig 1B). Cytological analysis showed the presence of clusters of relatively small cells, along the length of the anthers (compare Fig 2A and Fig 2B). This abnormal tissue bore a similarity to the transmitting tissue found in the centre of the style (Fig 2C). Furthermore, ovule-like protrusions were found at the base of the anthers (Fig 2D). Thus, tissues and organs resembing those normally found in the inner-most whorl of tomato flowers were present on the anthers of flowers grown under CMH32, suggesting that CMH32 induced a partial homeotic conversion from anthers to pistil.

**Fig 3:**
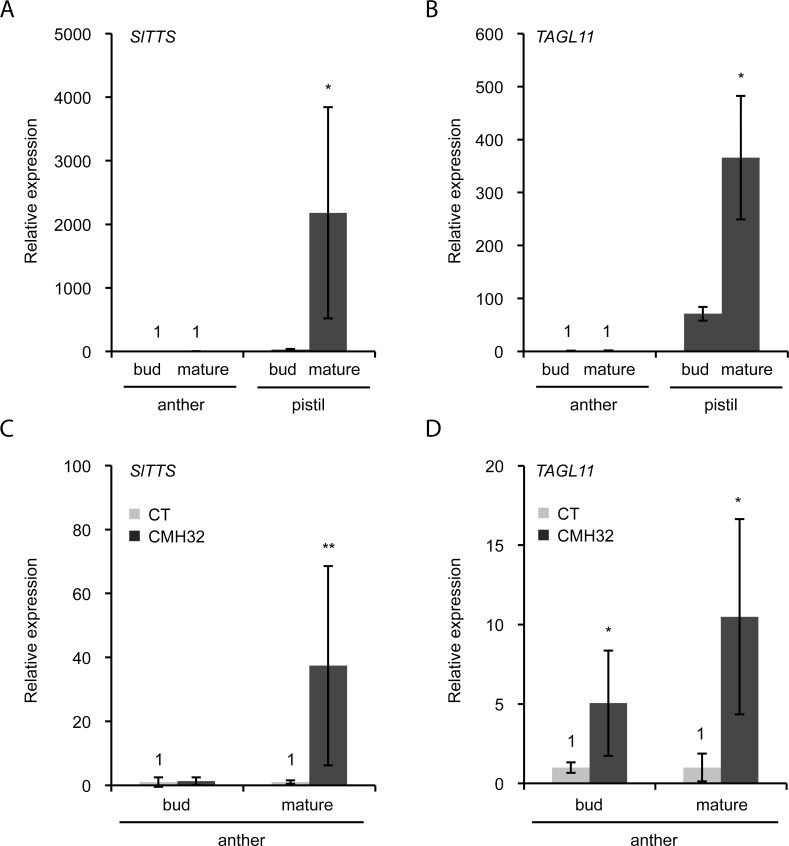
Relative expression of pistil marker genes in anthers and pistils of Red Setter grown under control conditions and continuous mild heat (CMH32). A, B, Relative expression of the *SlTTS* (A) and *TAGL11* (B) in anthers and pistils from 2-3mm flower buds and mature flowers (grown under control conditions). C, D, relative expression of *SlTTS* (C) and *TAGL11* (D) in anthers grown under control conditions (CT) and CMH32. Values represent the mean ± SE, with the mean expression in anthers (A, B) or under control conditions (C, D) at each developmental stage set to 1. *, significantly different from anther (A, B) or the control treatment (C, D), P<0.05; **, P<0.01; ***, P<0.001.

To independently confirm that the anthers gained pistil-like identity under CMH32, we harvested anthers and pistils from plants grown under control and CMH32 conditions and analyzed the expression of two pistil-specific genes. In tobacco, *TRANSMITTING TISSUE SPECIFIC* (*TTS*) is known to be expressed in the transmitting tissue of the style [32]. The tomato ortholog (*Solyc02g078100*), which we named *SlTTS*, was identified on the basis of sequence similarity. Gene expression analysis showed that its transcript indeed accumulated specifically in pistils, with strongest expression in mature flowers (Fig 3A). Similarly, we confirmed that the expression of the MADS-box gene *TOMATO AGAMOUS LIKE11* (*TAGL11*), involved in ovule development [33], was pistil specific (Fig 3B). Subsequent analysis showed that the anthers of plants grown under CMH32 had significantly higher expression of *SlTTS* and *TAGL11* then those from plants grown under control conditions (Fig 3C and 3D). This result supports the hypothesis that the observed anther deformations are homeotic conversions.

### Expression of B- and C-class genes under CMH32

**Fig 4:**
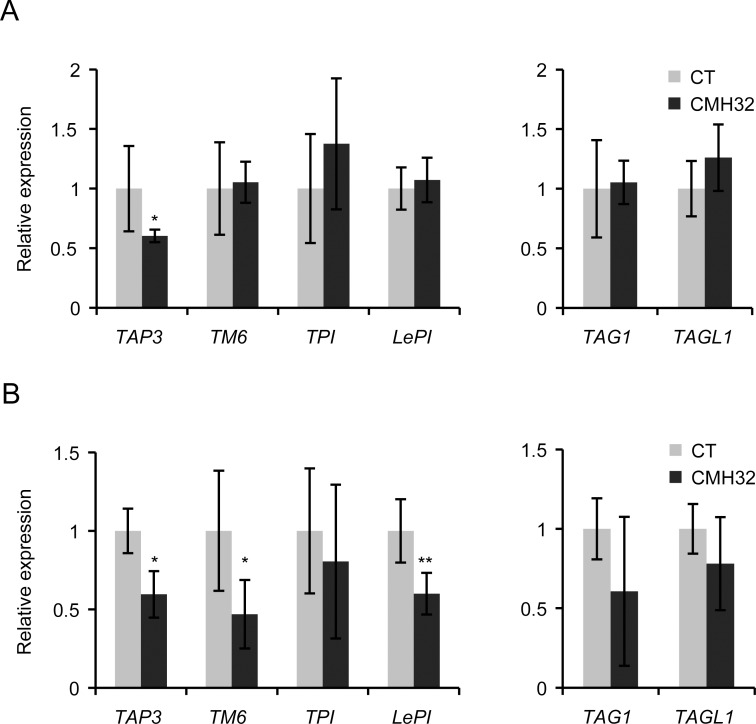
Relative expression of B- and C-class genes under control and continuous mild heat conditions (CMH32) in anthers of the tomato cultivar Red setter. A, expression of the B-class genes *TAP3*, *TM6*, *TPI* and *LePI* and the C-class genes *TAG1* and *TAGL1* in young anthers of 2–3 mm. B, gene expression in mature anthers. Values represent the mean ± SE, with the mean expression in the control condition set to 1. *, significantly different from the control treatment, P<0.05; **, P<0.01; ***, P<0.001.

**Fig 5:**
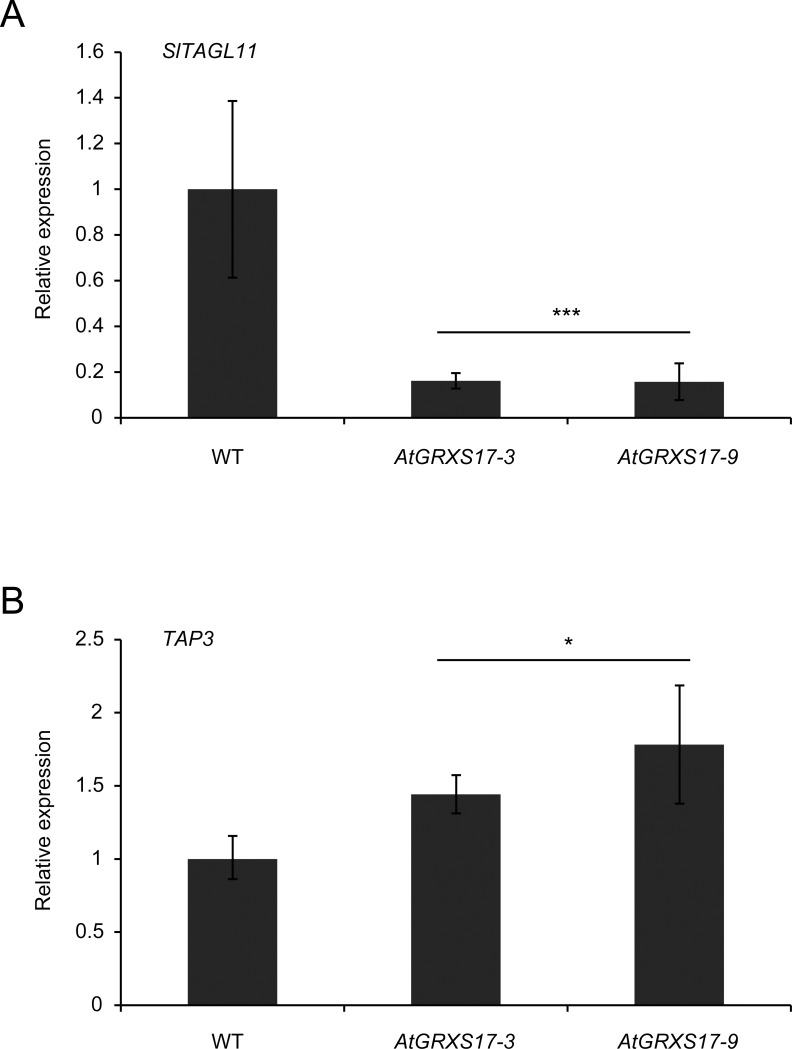
Relative expression of the pistil marker gene *TAGL11* and B-class gene *TAP3* in wild-type and *AtGRXS17*-overexpressing lines under continuous mild heat (CMH32) conditions. A, B, Relative expression of *TAGL11* (A) and *TAP3* (B) in young anthers of 2–3 mm in wild type (WT) and *AtGRXS17*-overexpressing lines grown under CMH32. Values represent the mean ± SE, with the mean expression in the wild type set to 1. *, significantly different from the wild type, P<0.05; ***, P<0.001.

Anther identity depends on activity of B- and C-class genes and a decreased ratio of B- to C-class gene expression may lead to pistil-like identity in the anthers [34]. Therefore, we analyzed expression of these classes of genes in anthers grown under control and CMH32 conditions. In core eudicots, the *APETALA3* (*AP3*) B-class gene lineage has diverged into two paralogous lineages: *euAP3* and *TOMATO MADS BOX GENE6* (*TM6*) [35]. While in Arabidopsis and Antirrhinum only the *euAP3* lineage has been retained [36], tomato has both lineages, i.e. *TOMATO AP3* (*TAP3*) and *TM6* [34]. A loss-of-function mutation in *TAP3* results in a homeotic conversion of stamen to pistil-like tissue, as well as conversion of petals to sepal-like structures [37]. Silencing of *TM6*, on the other hand, only leads to homeotic conversions of the stamen [34,37]. Similarly, tomato contains two PISTILLATA-type B-class genes, i.e. *Lycopersicum esculentum PISTILLATA* (*LePI*) and *TOMATO PISTILLATA* (*TPI*) [34,38]. Fig 4 shows that the relative expression of the B-class gene *TAP3* was already reduced in young anthers grown under CMH32, while in mature anthers also *TM6* and *LePI* were expressed significantly lower. The B-class gene *TPI* was not differentially expressed, and also expression of the C-class genes *TOMATO AGAMOUS1* (*TAG1*) and *TAG-LIKE1* (*TAGL1)* was not significantly affected by CMH32. Interestingly, the more heat tolerant *AtGRXS17*-overexpression lines (Fig 1C) not only showed markedly reduced ectopic expression of the pistil marker *TAGL11* in young anthers under CMH32 than the wild type, but also significantly higher *AP3* expression (Fig 5).

### Flower phenotype of a TM6-RNAi line under CMH30 and control conditions

To determine whether the observed down-regulation of B-class genes in anthers under CMH32 acted up- or downstream of the change in identity, we analysed a weak *TM6*-RNAi line [34]. As was previously described, this line showed only very few anther-to-pistil conversions when grown under control conditions (Table 2). However, when grown under CMH30, a temperature regime not high enough to cause homeotic conversions in the wild-type background, the frequency of anther-to-pistil related deformations in this line increased significantly (Table 2). At this temperature regime, most of the *TM6*-RNAi flowers had strong anther deformations, even stronger than those of wild-type plants under CMH32. The enhanced effect at CMH30 might be due to combined mild reductions in *TM6* and *AP3* expression, although activities of these genes are not dependent on each other [34].

**Table 2:** Presence of anther deformations, pollen germination rate and pollen number of wild-type Microtom and the *TM6*–RNAi line under control and CMH30 conditions.

	Anther deformations (%)		Pollen germination (%)		Pollen number (*10^3^)	
	CT	CMH30	CT	CMH30	CT	CMH30
WT	0 ± 0 ^A^ (32)	0 ± 0 ^A^ (73)	76.4 ± 9.6 ^A^ (26)	24.9 ± 19.4 ^B^ (12)	71.8 ± 25.4 ^A^ (19)	20.6 ± 10.8 ^B^ (23)
*TM6i6*	12.4 ± 7.5 ^A^ (80)	57.1 ± 14.1 ^B^ (82)	36.6 ± 15.5 ^B^ (22)	0 ± 0 ^C^ (8)	9.7 ± 6.5 ^BC^ (18)	3.9 ± 3.5 ^C^ (19)
Genotype	< 0.001 (11.634)		< 0.001 (55.951)		<0.001 (39.117)	
Treatment	< 0.01 (7.855)		< 0.001 (68.868)		<0.001 (15.042)	
Interaction	< 0.01 (8.554)		0.014 (6.453)		0.044 (4.219)	

Plants were grown under control temperatures (CT) or CMH30. Values are mean ± SD. The number of analysed flowers is indicated between brackets. Different capitals indicate significant differences between the treatment-genotype groups for the concerning trait, P<0.05. Significances of the main effects and their interaction according to the two-way analyses are indicated, with χ^2^ or F-values between brackets.

### Effect of reduced B-Class gene activity on pollen viability

To determine whether reduction of B-Class gene activity also affected pollen development, we analysed pollen number and pollen germination potential of the wild type and *TM6*-RNAi lines grown under control and CMH30. As expected, pollen number and viability was somewhat reduced by CMH30 in the wild type. Already under control conditions, *TM6*-RNAi plants produced significantly fewer pollen than the wild type, and the remaining pollen had lower viability (Table 2). Under CMH30, these characteristics were further affected, especially pollen viability. There was a significant interaction between genotype and treatment for both traits, in that the *TM6*-RNAi line responded singnificantly more strongly to the treatment than the wild type (Table 2). Taken together, our results show that an artificial decrease in *TM6* expression mimics/enhances high temperature-induced phenotypic defects in both, anther and pollen development.

## Discussion

In the current era of global warming, high temperature damage to plants is a increasingly frequent phenomenon, particularly around the reproductive period [39,40]. Heat-induced male infertility has been noted in a wide variety of angiosperms and a link between defects in pollen development and the function of sporophytic anther tissue under high temperature has been hypothesized. Data presented here suggest a mechanistic basis for this relation. Our results fit a model in which continuous mild heat represses B-class gene activity, which normally acts to direct stamen identity and thereby supports pollen development. Interestingly, gross morphological changes in stamen size and morphology have been noted to accompany reduction of pollen viability not only in the various tomato accessions in this study, but also in a number of other species [41–44]. Furthermore, closer inspection of a data set from [8] on transcriptomic changes in developing flower organs of barley under CMH revealed a similar down-regulation of B-class genes (data not shown). Together, these findings suggest that the model proposed may be more broadly applicable to flowering plants.

Support for our results comes from the tomato *stamenless* type of mutants mutant. The *sl* mutant has complete homeotic conversion of anthers to carpels (fused to the central carpel) and was recently found to be a *TAP3* mutant [37,45]. *sl-2* is likely to be allelic to *sl* [46], but has a weaker homeotic phenotype, analogous to the *TM6*-RNAi line used in our studies [37,47]. Interestingly, the flower phenotypes of these mutants are temperature dependend, as flower morphology and fertility of both can be rescued by growth at reduced temperatures and the anther and pollen defects of the *sl-2* mutant are strongly enhanced when the plants are grown at elevated temperature [45,47]. These findings are in accordance with the fact that *TAP3/TM6* gene expression is reduced by high temperature (Fig 4) and induced by low temperature treatment [48]. Thus, B-class gene activity seems to be negatively regulated by ambient temperature over a broad range of temperatures.

Studies in Arabidopsis have shown that expression of B-class genes is regulated by different pathways over the course of flower development. During the floral meristem patterning stage, their expression is induced by *LEAFY* and *APETALA1* [49,50]. At later stages of anther and pollen development, however, B-class gene expression is maintained by gibberellin (GA) via degradation of DELLA proteins, which act as repressors of the B-class MADS-box genes [50,51]. Notably, a GA-hyposensitive mutant in wheat has recently been shown to be hypersensitive to high temperature regarding seed set [52] and among rice cultivars, the ability to maintain pollen viability under high temperatures was shown to correlate with higher contents of GA [53].

Phenotypically, male infertility as caused by high growth temperature is very similar to that caused by drought during flower development, including abberent development of the tapetum [1,24]. In Arabidopsis, it has been shown that *AP3* gene expression is also reduced under drought [54], which may mean that the similarity extends towards the underlying molecular defect. Interestingly, homeotic anther conversion has also been described for a cytoplasmic male sterile (CMS) *Brassica napus* line and expression of the B class genes was strongly reduced in the CMS line [55]. The reason for the anther phenotype is unknown, but as the CMS is caused by mitochondrial dysfunction, it has been suggested to be related to higher reactive oxygen species (ROS) production [56]. Because high temperature is known to affect ROS levels, it is tempting to hypothesize that this represents the initial defect in this condition as well. This would agree to our finding that overexpression of a glutaredoxin gene improves anther and pollen development and *AP3* gene expression under high temperature. Future studies combining manipulations of B-class gene activity, GA signaling strength and ROS levels should shed light on this.

## References

[1] Parish RW, Phan HA, Iacuone S, Li SF. Tapetal development and abiotic stress: a centre of vulnerability. Funct Plant Biol. 2012; 39(7):553–9. 10.1071/FP12090 32480807

[2] Bokszczanin KL, Solanaceae Pollen Thermotolerance Initial Training Network (SPOT-ITN) Consortium, Fragkostefanakis S. Perspectives on deciphering mechanisms underlying plant heat stress response and thermotolerance. Front Plant Sci. 2013; 4:315 10.3389/fpls.2013.00315 23986766 PMC3750488

[3] IPCC. Climate change 2014: impacts, adaptation and vulnerability. Part A: Global and Sectoral Apsects. Working group II contribution to the Fifth Assessment Report of the Intergovernmental Panel on Climate Change. Field CB, Barros VR, Dokken DJ, Mach KJ, Mastrandrea MD, Bilir TE, et al., editors. Cambridge, United Kingdom and New York, NY, USA: Cambridge University Press; 2014. 1132 p.

[4] Hedhly A, Hormaza JI, Herrero M. Global warming and sexual plant reproduction. Trends Plant Sci. 2009; 14(1):30–6. 10.1016/j.tplants.2008.11.001 19062328

[5] Zinn KE, Tunc-Ozdemir M, Harper JF. Temperature stress and plant sexual reproduction: uncovering the weakest links. J Exp Bot. 2010; 61(7):1959–68. 10.1093/jxb/erq053 20351019 PMC2917059

[6] Sato S, Peet MM, Thomas JF. Physiological factors limit fruit set of tomato (*Lycopersicon esculentum* Mill.) under chronic, mild heat stress. Plant Cell Environ. 2000; 23(7):719–26.

[7] Ku SJ, Yoon H, Suh HS, Chung YY. Male-sterility of thermosensitive genic male-sterile rice is associated with premature programmed cell death of the tapetum. Planta. 2003; 217(4):559–65. 10.1007/s00425-003-1030-7 12692728

[8] Oshino T, Abiko M, Saito R, Ichiishi E, Endo M, Kawagishi-Kobayashi M, et al Premature progression of anther early developmental programs accompanied by comprehensive alterations in transcription during high-temperature injury in barley plants. Mol Genet Genomics. 2007; 278(1):31–42. 10.1007/s00438-007-0229-x 17429694

[9] Frank G, Pressman E, Ophir R, Althan L, Shaked R, Freedman M, et al Transcriptional profiling of maturing tomato (*Solanum lycopersicum* L.) microspores reveals the involvement of heat shock proteins, ROS scavengers, hormones, and sugars in the heat stress response. J Exp Bot. 2009; 60(13):3891–908. 10.1093/jxb/erp234 19628571 PMC2736902

[10] Firon N, Shaked R, Peet MM, Pharr DM, Zamski E, Rosenfeld K, et al Pollen grains of heat tolerant tomato cultivars retain higher carbohydrate concentration under heat stress conditions. Sci Hort. 2006; 109(3):212–7.

[11] Pressman E, Peet MM, Pharr DM. The effect of heat stress on tomato pollen characteristics is associated with changes in carbohydrate concentration in the developing anthers. Ann Bot. 2002; 90(5):631–6. 10.1093/aob/mcf240 12466104 PMC4240456

[12] Zhang XW, Li JP, Liu AL, Zou J, Zhou XY, Xiang JH, et al Expression profile in rice panicle: Insights into heat response mechanism at reproductive stage. PloS ONE. 2012; 7(11): e49652 10.1371/journal.pone.0049652 23155476 PMC3498232

[13] Song JJ, Nada K, Tachibana S. Suppression of S-adenosylmethionine decarboxylase activity is a major cause for high-temperature inhibition of pollen germination and tube growth in tomato (*Lycopersicon esculentum* Mill.). Plant Cell Physiol. 2002; 43(6):619–27. 12091715 10.1093/pcp/pcf078

[14] Zhang HQ, Croes AF. Protection of pollen germination from adverse temperatures—a possible role for proline. Plant Cell Environ. 1983; 6(6):471–6.

[15] Singh I, Shono M. Physiological and molecular effects of 24-epibrassinolide, a brassinosteroid on thermotolerance of tomato. Plant Growth Regul. 2005; 47(2–3):111–9.

[16] Endo M, Tsuchiya T, Hamada K, Kawamura S, Yano K, Ohshima M, et al High temperatures cause male sterility in rice plants with transcriptional alterations during pollen development. Plant Cell Physiol. 2009; 50(11):1911–22. 10.1093/pcp/pcp135 19808807

[17] Chapman GP. The Tapetum. Int Rev Cytol. 1987; 107:111–25.

[18] Ahmed FE, Hall AE, Demason DA. Heat injury during floral development in cowpea (*Vigna unguiculata*, Fabaceae). Am J Bot. 1992; 79(7):784–91.

[19] Harsant J, Pavlovic L, Chiu G, Sultmanis S, Sage TL. High temperature stress and its effect on pollen development and morphological components of harvest index in the C-3 model grass *Brachypodium distachyon*. J Exp Bot. 2013; 64(10):2971–83. 10.1093/jxb/ert142 23771979 PMC3697958

[20] Abiko M, Akibayashi K, Sakata T, Kimura M, Kihara M, Itoh K, et al High-temperature induction of male sterility during barley (*Hordeum vulgare* L.) anther development is mediated by transcriptional inhibition. Sex Plant Reprod. 2005; 18(2):91–100.

[21] Iwahori S. High temperature injuries in tomato. IV. Development of normal flower buds and morphological abnormalities of flower buds treated with high temperature. J Jpn Soc Hortic Sci. 1965; 34(1):33–41.

[22] Saini HS, Sedgley M, Aspinall D. Developmental anatomy in wheat of male-sterility induced by heat-stress, water deficit or abscisic-acid. Aust J Plant Physiol. 1984; 11(4):243–53.

[23] Suzuki K, Takeda H, Tsukaguchi T, Egawa Y. Ultrastructural study on degeneration of tapetum in anther of snap bean (*Phaseolus vulgaris* L.) under heat stress. Sex Plant Reprod. 2001; 13(6):293–9.

[24] De Storme N, Geelen D. The impact of environmental stress on male reproductive development in plants: biological processes and molecular mechanisms. Plant Cell Environ. 2014; 37(1):1–18. 10.1111/pce.12142 23731015 PMC4280902

[25] Ng M, Yanofsky MF. Function and evolution of the plant MADS-box gene family. Nature Rev Genet. 2001; 2(3):186–95. 10.1038/35056041 11256070

[26] Coen ES, Meyerowitz EM. The war of the whorls: genetic interactions controlling flower development. Nature. 1991; 353(6339):31–7. 10.1038/353031a0 1715520

[27] Becker A, Theissen G. The major clades of MADS-box genes and their role in the development and evolution of flowering plants. Mol Phylogenet Evol. 2003; 29(3):464–89. 14615187 10.1016/s1055-7903(03)00207-0

[28] Wu Q, Lin J, Liu JZ, Wang X, Lim W, Oh M, et al Ectopic expression of *Arabidopsis* glutaredoxin *AtGRXS17* enhances thermotolerance in tomato. Plant Biotech J. 2012; 10(8):945–55. 10.1111/j.1467-7652.2012.00723.x 22762155

[29] Pino-Nunes LE, Figueira AVO, Tulmann Neto A, Zsögön A, Piotto FA, Silva JA, et al Induced mutagenesis and natural genetic variation in tomato 'Micro-Tom'. Acta Hortic. 2009; 821:63–72.

[30] Rieu I, Powers SJ. Real-time quantitative RT-PCR: Design, calculations, and statistics. Plant Cell. 2009; 21(4):1031–3. 10.1105/tpc.109.066001 19395682 PMC2685626

[31] Peet MM, Sato S, Gardner RG. Comparing heat stress effects on male-fertile and male-sterile tomatoes. Plant Cell Environ. 1998; 21(2):225–31.

[32] Cheung AY, May B, Kawata EE, Gu Q, Wu HM. Characterization of cDNAs for stylar transmitting tissue-specific proline-rich proteins in tobacco. Plant J. 1993; 3(1):151–60. 8401601

[33] Victoria BM, Claudia B, Cecilia D, Mauricio HC, Silvana BB, Estela VM, et al MADS-box genes expressed during tomato seed and fruit development. Plant Mol Biol. 2003; 52(4):801–15. 13677468 10.1023/a:1025001402838

[34] de Martino G, Pan I, Emmanuel E, Levy A, Irish VF. Functional analyses of two tomato *APETALA3* genes demonstrate diversification in their roles in regulating floral development. Plant Cell. 2006; 18(8):1833–45. 10.1105/tpc.106.042978 16844904 PMC1533988

[35] Kramer EM, Dorit RL, Irish VF. Molecular evolution of genes controlling petal and stamen development: Duplication and divergence within the *APETALA3* and *PISTILLATA* MADS-box gene lineages. Genetics. 1998; 149(2):765–83. 9611190 10.1093/genetics/149.2.765 PMC1460198

[36] Bowman JL, Smyth DR, Meyerowitz EM. Genes directing flower development in *Arabidopsis*. Plant Cell. 1989; 1(1):37–52. 10.1105/tpc.1.1.37 2535466 PMC159735

[37] Quinet M, Bataille G, Dobrev PI, Capel C, Gomez P, Capel J, et al Transcriptional and hormonal regulation of petal and stamen development by *STAMENLESS*, the tomato (*Solanum lycopersicum* L.) orthologue to the B-class *APETALA3* gene. J Exp Bot. 2014; 65(9):2243–56. 10.1093/jxb/eru089 24659487 PMC4036497

[38] Mazzucato A, Olimpieri I, Siligato F, Picarella ME, Soressi GP. Characterization of genes controlling stamen identity and development in a parthenocarpic tomato mutant indicates a role for the *DEFICIENS* ortholog in the control of fruit set. Physiol Plant. 2008; 132(4):526–37. 10.1111/j.1399-3054.2007.01035.x 18334005

[39] Lobell DB, Gourdji SM. The influence of climate change on global crop productivity. Plant Physiol. 2012; 160(4):1686–97. 10.1104/pp.112.208298 23054565 PMC3510102

[40] Gourdji SM, Sibley AM, Lobell DB. Global crop exposure to critical high temperatures in the reproductive period: historical trends and future projections. Environ Res Lett. 2013; 8(2).

[41] Sakata T, Takahashi H, Nishiyama I, Higashitani A. Effects of high temperature on the development of pollen mother cells and microspores in barley *Hordeum vulgare* L. J Plant Res. 2000; 113(1112):395–402.

[42] Porch TG, Jahn M. Effects of high-temperature stress on microsporogenesis in heat-sensitive and heat-tolerant genotypes of *Phaseolus vulgaris*. Plant Cell Environ. 2001; 24(7):723–31.

[43] Sakata T, Oshino T, Miura S, Tomabechi M, Tsunaga Y, Higashitani N, et al Auxins reverse plant male sterility caused by high temperatures. Proc Natl Acad Sci U S A. 2010; 107(19):8569–74. 10.1073/pnas.1000869107 20421476 PMC2889339

[44] Min L, Zhu LF, Tu LL, Deng FL, Yuan DJ, Zhang XL. Cotton *GhCKI* disrupts normal male reproduction by delaying tapetum programmed cell death via inactivating starch synthase. Plant J. 2013; 75(5):823–35. 10.1111/tpj.12245 23662698

[45] Gomez P, Jamilena M, Capel J, Zurita S, Angosto T, Lozano R. *Stamenless*, a tomato mutant with homeotic conversions in petals and stamens. Planta. 1999; 209(2):172–9. 10.1007/s004250050619 10436218

[46] Nash AF, Gardner RG, Henderson WR. Evaluation of allelism and seed set of 8 *stamenless* tomato mutants. Hortscience. 1985; 20(3):440–2.

[47] Sawhney VK, Greyson RI. Morphogenesis of *stamenless-2* mutant in tomato. 1. Comparative description of flowers and ontogeny of stamens in normal and mutant plants. Am J Bot. 1973; 60(6):514–23.

[48] Lozano R, Angosto T, Gomez P, Payan C, Capel J, Huijser P, et al Tomato flower abnormalities induced by low temperatures are associated with changes of expression of MADS-Box genes. Plant Physiol. 1998; 117(1):91–100. 9576778 10.1104/pp.117.1.91 PMC35026

[49] Winter CM, Austin RS, Blanvillain-Baufume S, Reback MA, Monniaux M, Wu MF, et al *LEAFY* target genes reveal floral regulatory logic, cis motifs, and a link to biotic stimulus response. Dev Cell. 2011; 20(4):430–43. 10.1016/j.devcel.2011.03.019 21497757

[50] Weigel D, Alvarez J, Smyth DR, Yanofsky MF, Meyerowitz EM. *LEAFY* controls floral meristem identity in Arabidopsis. Cell. 1992; 69(5):843–59. 1350515 10.1016/0092-8674(92)90295-n

[51] Yu H, Ito T, Zhao YX, Peng JR, Kumar PP, Meyerowitz EM. Floral homeotic genes are targets of gibberellin signaling in flower development. Proc Natl Acad Sci U S A. 2004; 101(20):7827–32. 10.1073/pnas.0402377101 15128937 PMC419691

[52] Alghabari F, Lukac M, Jones HE, Gooding MJ. Effect of Rht alleles on the tolerance of wheat grain set to high temperature and drought stress during booting and anthesis. J Agron Crop Sci. 2014; 200(1):36–45.

[53] Tang RS, Zheng JC, Jin ZQ, Zhang D, Huang H, Chen LG. Possible correlation between high temperature-induced floret sterility and endogenous levels of IAA, GAs and ABA in rice (*Oryza sativa* L.). Plant Growth Regul. 2008; 54(1):37–43.

[54] Su Z, Ma X, Guo HH, Sukiran NL, Guo B, Assmann SM, et al Flower development under drought stress: Morphological and transcriptomic analyses reveal acute responses and long-term acclimation in Arabidopsis. Plant Cell. 2013; 25(10):3785–807. 10.1105/tpc.113.115428 24179129 PMC3877795

[55] Teixeira RT, Farbos I, Glimelius K. Expression levels of meristem identity and homeotic genes are modified by nuclear-mitochondrial interactions in alloplasmic male-sterile lines of *Brassica napus*. Plant J. 2005; 42(5):731–42. 10.1111/j.1365-313X.2005.02407.x 15918886

[56] Horn R, Gupta KJ, Colombo N. Mitochondrion role in molecular basis of cytoplasmic male sterility. Mitochondrion. 2014; 19:198–205. 10.1016/j.mito.2014.04.004 24732436

